# Identification and Characterization of SMARCAL1 Protein Complexes

**DOI:** 10.1371/journal.pone.0063149

**Published:** 2013-05-09

**Authors:** Rémy Bétous, Gloria G. Glick, Runxiang Zhao, David Cortez

**Affiliations:** Department of Biochemistry, Vanderbilt University School of Medicine, Nashville, Tennessee, United States of America; Universita’ di Milano, Italy

## Abstract

SMARCAL1 is an ATPase in the SNF2 family that functions at damaged replication forks to promote their stability and restart. It acts by translocating on DNA to catalyze DNA strand annealing, branch migration, and fork regression. Many SNF2 enzymes work as motor subunits of large protein complexes. To determine if SMARCAL1 is also a member of a protein complex and to further understand how it functions in the replication stress response, we used a proteomics approach to identify interacting proteins. In addition to the previously characterized interaction with replication protein A (RPA), we found that SMARCAL1 forms complexes with several additional proteins including DNA-PKcs and the WRN helicase. SMARCAL1 and WRN co-localize at stalled replication forks independently of one another. The SMARCAL1 interaction with WRN is indirect and is mediated by RPA acting as a scaffold. SMARCAL1 and WRN act independently to prevent MUS81 cleavage of the stalled fork. Biochemical experiments indicate that both catalyze fork regression with SMARCAL1 acting more efficiently and independently of WRN. These data suggest that RPA brings a complex of SMARCAL1 and WRN to stalled forks, but that they may act in different pathways to promote fork repair and restart.

## Introduction

The stabilization, repair, and restart of stalled replication forks are necessary to accurately complete DNA replication. Fork stalling is common due to damaged DNA templates, insufficient nucleotide precursors, collisions between the replisome and transcriptional complexes, and difficult to replicate genomic regions. These circumstances lead to an uncoupling of enzymatic activities at the fork, the appearance of excess single-stranded DNA (ssDNA), and the activation of a DNA damage response controlled by the ATR kinase [1].

Two proteins that are recruited to stalled forks are SMARCAL1 and WRN. Both bind to the ssDNA binding protein RPA and are required to maintain genome integrity during S-phase [2]–[10]. Both are also ATR substrates [2], [11]–[13]. Furthermore, bi-allelic, loss of function mutations in both genes cause diseases with pleiotropic phenotypes. *SMARCAL1* mutations cause Schimke Immunoosseous Dysplasia (SIOD) [14]. SIOD patients suffer from bone growth defects, immunodeficiencies, renal failure, other varied symptoms, and are predisposed to cancer [15], [16]. Onset of symptoms varies from *in utero* to early adolescence. *WRN* mutations cause Werners Syndrome [17]. This disease is characterized by growth defects at the time of puberty, premature aging, and increased cancer risk.

Both SMARCAL1 and WRN bind directly to DNA. SMARCAL1 functions as an annealing helicase that can promote the annealing of two DNA strands [18]. It also catalyzes branch migration and fork regression [19], [20]. It lacks helicase activity at least on typical test substrates. WRN has both helicase and exonuclease activities [21]. Its helicase activity can also promote fork regression [22].

SMARCAL1 is a member of the SNF2 family of ATPases [23]. Many of these proteins act as part of larger protein complexes. To understand if SMARCAL1 acts as part of a complex or has protein interaction partners that regulate its activity in addition to RPA, we undertook a proteomics approach to identify interacting proteins. This approach identified several associated proteins including WRN. A previous publication also reported WRN in a mass spectrometry screen for SMARCAL1 interacting proteins, although no validation or functional data was reported [3]. Here we describe our characterization of the SMARCAL1-WRN interaction and its functional significance.

## Materials and Methods

### Cell Culture

U2OS, HEK293T, and HeLa cells were obtained from ATCC and maintained in DMEM with 7.5% FBS. siRNA transfections were performed using either HiPerfect (Qiagen) or Dharmafect 1 (Dharmacon) at a final siRNA concentration of 10 µM. siRNAs were purchased from Dharmacon.

### Immunoblotting, Immunofluorescence, and Antibodies

Rabbit polyclonal SMARCAL1 909 antibody was described previously [2]. Additional antibodies include: RPA32, (clone 9H8, Abcam); γH2AX (clone JBW301 Upstate Biotechnology); and Flag M2 (Sigma); TOPO-1 (Abcam); TOPO-II alpha (Bethyl); SPT16 (H300, Santa Cruz); DNA-PKcs (Santa Cruz); WRN (Novus, NB100-471 for immunoblots and Bethyl, A300-239 for immunoprecipitation); Quantitative immunoblotting was performed using an Odyssey instrument. For immunofluorescence, cells were fixed with 3% formaldehyde and permeabilized with 0.5% triton X-100.

### Construction of SMARCAL1 Expression Vectors

All expression vectors were made using the Gateway cloning system. The wild-type and Δ34 SMARCAL1 vectors were described previously [2]. The SMARCAL1-Δ34 RPA-BD1 expression vector was created by inserting DNA sequences encoding the following peptide upstream of the first SMARCAL1 codon into the SMARCAL1-Δ34 vector: DFTADDLEEWFALAS. This peptide is derived from human ATRIP and optimized to improve binding affinity to RPA70N (data not shown). The SMARCAL1-Δ34 RPA-BD2 expression vector was created by inserting the first 107 amino acids of human ATRIP containing the RPA70N binding domain upstream of the first SMARCAL1 codon into the SMARCAL1-Δ34 vector.

### Immunoprecipitations and Mass Spectrometry

Both SMARCAL1 and WRN immunoprecipitations (IP) were performed using nuclear extracts (NE) from Hela-S3 cells using the same procedure as previously described [2].

### Fork Regression Assay

Flag-SMARCAL1 was purified from baculovirus-infected cells essentially as described previously [19]. WRN was a kind gift of Patricia Opresko, University of Pittsburgh. Supplemental Table S1 lists the oligonucleotide sequences. Oligonucleotides were end-labeled with [γ-^32^]-ATP and T4 polynucleotide kinase (NEB), and purified through a G25 column (GE healthcare). To prepare the fork regression substrate, labeled and unlabeled leading or lagging parental strands were annealed with their corresponding nascent strands in SSC buffer (15 mM NaCitrate, pH = 7.0, 150 mM NaCl) in a PCR machine. Then 0.75 µM of labeled and 1.1 µM of unlabeled DNA intermediates were incubated in annealing buffer (40 mM Tris (pH = 7.5), 20 mM KCl, 5 mM MgCl_2_, 100 µg/ml BSA, and 2 mM DTT) for 30 min at 37°C. Substrates from the annealing reactions were separated in a 5% polyacrylamide gel, excised from the gel and electro-eluted in 0.25X TBE. The DNA substrates were concentrated using a vacuum concentrator and stored at −80°C. 3 nM substrate, 6 nM RPA, and the indicated concentrations of SMARCAL1 and WRN were incubated in reaction buffer (40 mM Tris (pH = 7.5), 100 mM KCl, 5 mM MgCl_2_, 100 µg/ml BSA, 2 mM ATP and 2 mM DTT) for 20 min at 30°C. The reactions were terminated by the addition of 3X stop buffer (0.9% SDS, 50 mM EDTA, 40% glycerol, 0.1% bromophenol blue, and 0.1% xylene cyanol). Samples were loaded into 8% polyacrylamide 1X TBE gels (8×8.5 cm, 1 mm thick), and subjected to electrophoresis in 1X TBE for 90 min at 80 V at room temperature. The gels were dried and quantified using a Molecular Imager FX (Bio Rad).

## Results

### Identification of SMARCAL1 Interacting Proteins

**Figure 1:**
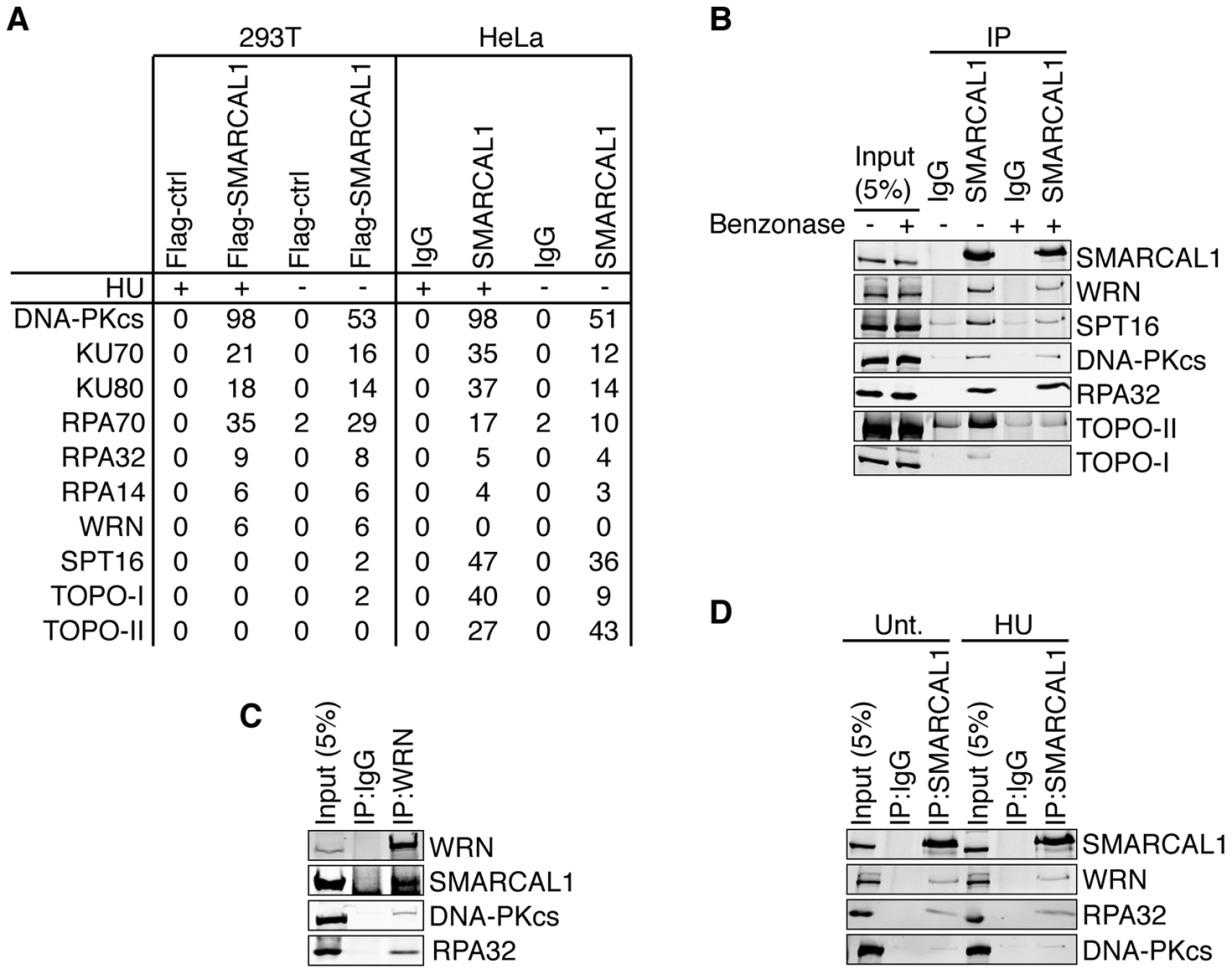
Identification of SMARCAL1 interacting proteins by mass spectrometry. (A) 293T cells were transfected with a Flag-SMARCAL1 expression vector or an empty vector control. Cells were harvested, lysed and Flag antibody conjugated beads were used to immunopurify SMARCAL1. HeLa cells expressing endogenous SMARCAL1 were harvested, lysed, and SMARCAL1-909 antibody or IgG control antibody were used for immunopurification. Proteins were eluted from beads with either the Flag or SMARCAL1-909 peptides and subjected to 2D-LC-tandem mass spectrometry. Where indicated, the cells were treated with 2 mM HU for 16 h prior to harvesting. The number of peptides identified in each purification is reported. (B–D) HeLa nuclear extracts were prepared. Control IgG, SMARCAL1-909 antibody, or WRN antibody immunoprecipitates (IP) as indicated were separated by SDS-PAGE and immunoblotted with the indicated antibodies. Where indicated the nuclear extracts were treated with benzonase prior to the immunoprecipitation. In (D) the cells were either mock treated (Unt.) or incubated with 2 mM HU for 16 h prior to harvesting.

To identify SMARCAL1 interacting proteins we used two proteomic approaches. First, SMARCAL1 was immunopurified from HeLa cell extracts using an antibody (909) to a C-terminal peptide. Second, Flag-HA-SMARCAL1 was purified from HEK293T cells using tandem affinity purification with antibodies to the Flag and HA epitopes. Both purifications were performed from both untreated and cells treated with hydroxyurea (HU) for 16 h. Protein complexes were then examined by two-dimensional liquid chromatography coupled tandem mass spectrometry. These experiments identified several proteins in the SMARCAL1 purifications including DNA-PKcs, KU70, KU80, RPA70, RPA32, RPA14, WRN, SPT16, TOPO-I, and TOPO-II (Figure 1A). The differences in the proteins identified between the HeLa and HEK293T cell results were not reproducible since co-immunoprecipitations could be verified in both cell types (see below).

To validate that these proteins were indeed complexed with SMARCAL1 we performed SMARCAL1 immunoprecipitations from HeLa cell nuclear extracts followed by immunoblotting with appropriate antibodies. WRN, SPT16, DNA-PKcs, TOPO-I, and TOPO-II were all found in the SMARCAL1 immunoprecipitates (Figure 1B). Pre-incubation of the protein lysates with the benzonase nuclease largely eliminated the co-immunoprecipitation with both TOPO-I and TOPO-II suggesting those interactions are mediated by DNA. However, most of the interaction with WRN, DNA-PKcs and SPT16 were retained suggesting that SMARCAL1 and these proteins form complexes independently of DNA. To confirm that SMARCAL1 and WRN do interact, we performed a reciprocal co-immunoprecipitation using antibodies to WRN (Figure 1C). We found SMARCAL1 in the WRN immunoprecipitate confirming the protein complex. WRN also interacted with DNA-PKcs and RPA in these experiments as expected from previous reports [7], [24]–[26]. Furthermore, the SMARCAL1 interaction with WRN, RPA, and DNA-PKcs was not changed by treating cells with HU (Figure 1D), suggesting that these interactions are not regulated by replication stress.

### SMARCAL1 Co-localizes with WRN to Stalled Replication Forks

**Figure 2:**
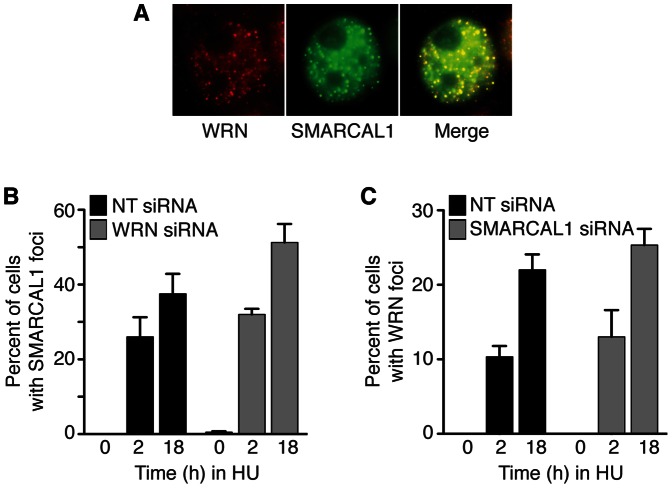
SMARCAL1 and WRN co-localize at stalled replication forks. (A) HeLa cells expressing GFP-WRN were treated with 2 mM HU, fixed, and stained with antibodies to SMARCAL1. (B) HeLa cells were transfected with non-targeting (NT) or WRN siRNAs then treated with 2 mM HU for the indicated times. Cells were fixed and stained with antibodies to SMARCAL1. (C) GFP-WRN expressing HeLa cells were transfected with NT or SMARCAL1 siRNAs, treated with 2 mM HU for the indicated times, fixed, and imaged for WRN. No significant difference is observed between the NT and SMARCAL1 siRNA samples.

Both SMARCAL1 and WRN are known to be diffusely localized in the nucleus in unstressed cells and localize to stalled replication forks in cells treated with DNA damage or replication stress agents [2], [8]. To confirm that they co-localize, we treated cells with HU and examined whether endogenous WRN would co-localize with GFP-SMARCAL1. As expected, we found significant co-localization within intra-nuclear foci (Figure 2A). WRN has been reported to be required for RPA localization to stalled forks in response to HU [27]. Since SMARCAL1 localization is dependent on RPA, we tested whether WRN depletion had any effect on the ability of SMARCAL1 to localize to stalled replication forks. Treating cells with WRN siRNA did not prevent SMARCAL1 from localizing to foci (Figure 2B). Likewise, treating cells with SMARCAL1 siRNA did not affect WRN localization (Figure 2C). If anything, the localization of each protein to foci was slightly increased in the absence of the binding partner perhaps due to increased DNA damage in cells after their depletion (see below). Thus, SMARCAL1 and WRN localize to stalled forks independently of one another.

### RPA Bridges the SMARCAL1-WRN Interaction

**Figure 3:**
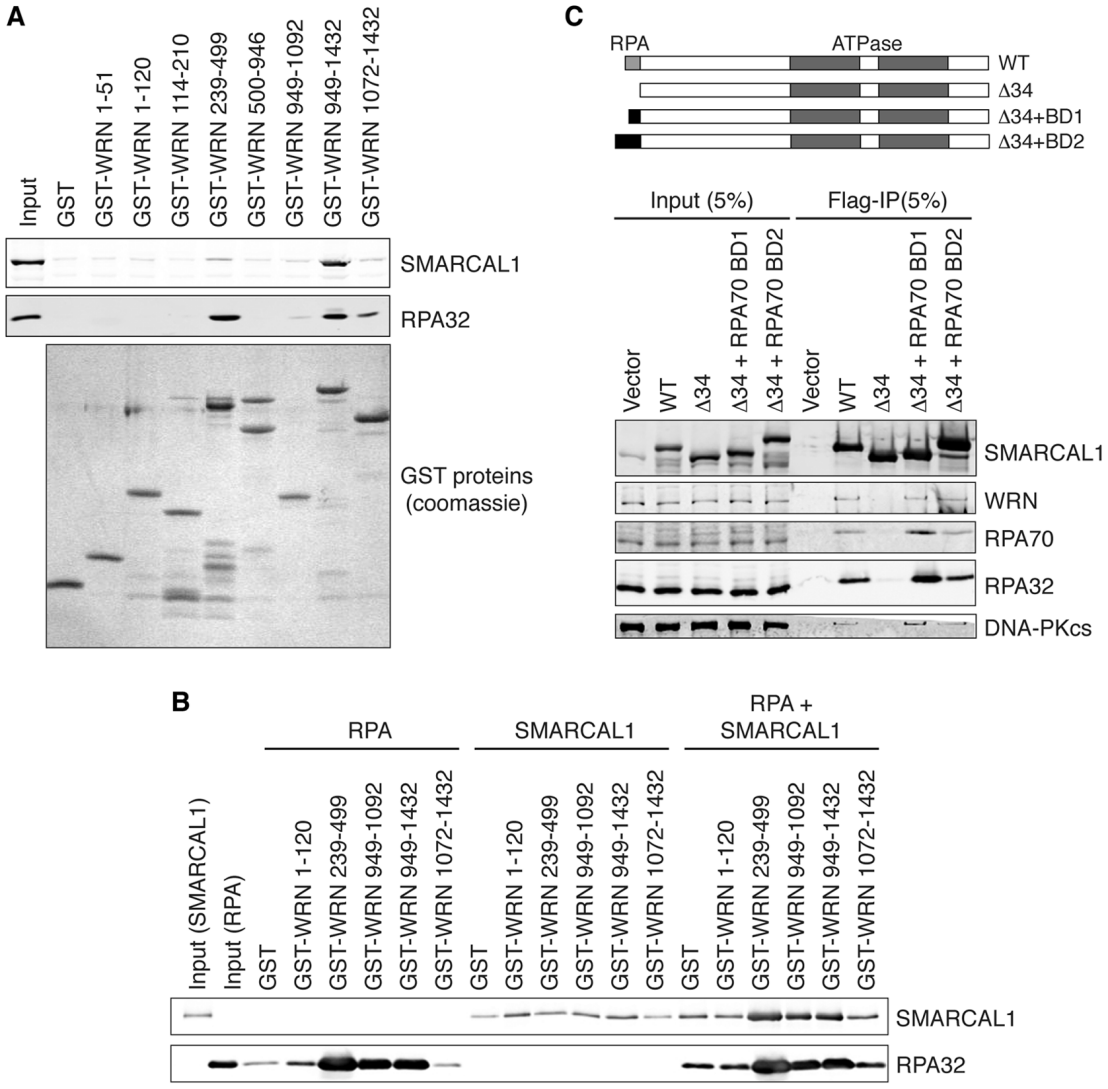
RPA acts as a scaffold to mediate the SMARCAL1-WRN interaction. (A) GST-WRN proteins bound to glutathione beads were incubated with HeLa nuclear extracts. After extensive washing, bound proteins were separated by SDS-PAGE and immunoblotted with antibodies to SMARCAL1 or RPA32. A coomassie stained gel was also prepared to document the amount of GST proteins added to the lysates. (B) Recombinant RPA, SMARCAL1, or both proteins were incubated with GST-WRN fragments bound to glutathione beads. After washing, bound proteins were separated by SDS-PAGE and immunoblotted with the indicated antibodies. (C) Flag immunoprecipitates from 293T cell lysates after transfection with Flag-SMARCAL1 wild-type (WT) or mutant expression vectors were separated by SDS-PAGE and immunoblotted with the indicated antibodies.

The binding site for SMARCAL1 on RPA is in the winged helix domain of RPA32 [2], [3]. WRN is reported to bind to a region of RPA70 containing amino acids 100–300 [25], [26]. Since it is possible for both SMARCAL1 and WRN to bind one molecule of RPA at the same time we asked whether RPA bridges the interaction between SMARCAL1 and WRN that we observed by co-immunoprecipitation. First, we incubated purified WRN protein fragments fused to GST with nuclear extracts, purified the bound proteins, and immunoblotted with antibodies to RPA and SMARCAL1. Interactions with both RPA and SMARCAL1 were seen with fragments containing amino acids 239–499 and 949–1432 (Figure 3A). These are the same fragments previously observed to bind to RPA [25]. This result suggests that either SMARCAL1 and RPA bind to the same surfaces on WRN, or that RPA bridges the interaction between WRN.

Next we incubated purified SMARCAL1 and RPA with these same WRN fragments. Again the major interaction surfaces for RPA were between amino acids 239–499 and 949–1432 although even the shorter 949–1092 WRN fragment also showed significant binding to RPA in this assay (Figure 3B). In the absence of RPA, only background levels of SMARCAL1 binding similar to the GST control were observed. However, when both RPA and SMARCAL1 were included in the pull-down assay, significant levels of SMARCAL1 were found associated with the same three GST-WRN fragments that bound to RPA in the absence of SMARCAL1. These results indicate that RPA acts as a scaffold to bridge the SMARCAL1-WRN interaction. The increased interactions of SMARCAL1 and RPA with the 239–499 and 949–1092 WRN fragments in this experiment compared to the pull-downs shown in Fig. 3A may be due to the presence of competing proteins in the nuclear extracts.

Finally, as a further test of this hypothesis, we analyzed the ability of several SMARCAL1 mutants to form a complex with WRN and RPA. Flag-SMARCAL1 wild-type (WT) was compared to Flag-SMARCAL1-Δ34 which lacks the RPA32 binding surface [2]. As expected if RPA is required for the complex to form, we failed to observe co-immunoprecipitation of the SMARCAL1-Δ34 protein with WRN (Figure 3C). We then replaced the N-terminal RPA32 interacting surface of SMARCAL1 with two RPA70N binding domains derived from the ATR-interacting protein ATRIP [28], [29]. BD1 is an optimized ATRIP peptide that has low micromolar affinity for RPA. BD2 is a larger peptide containing the first 107 amino acids of ATRIP that binds RPA70N. Fusing these RPA70N binding domains onto the SMARCAL1-Δ34 protein restored its ability to co-immunoprecipitate with RPA and WRN consistent with the bridging model for these interactions. Interestingly, in these experiments we also observed a similar interaction profile for DNA-PKcs with SMARCAL1 suggesting that this interaction is also a result of an RPA scaffold. DNA-PKcs was previously shown to interact with RPA70 although the exact interacting surface has not been identified [30].

### SMARCAL1 and WRN may Act Independently to Maintain Genome Integrity during DNA Replication

**Figure 4:**
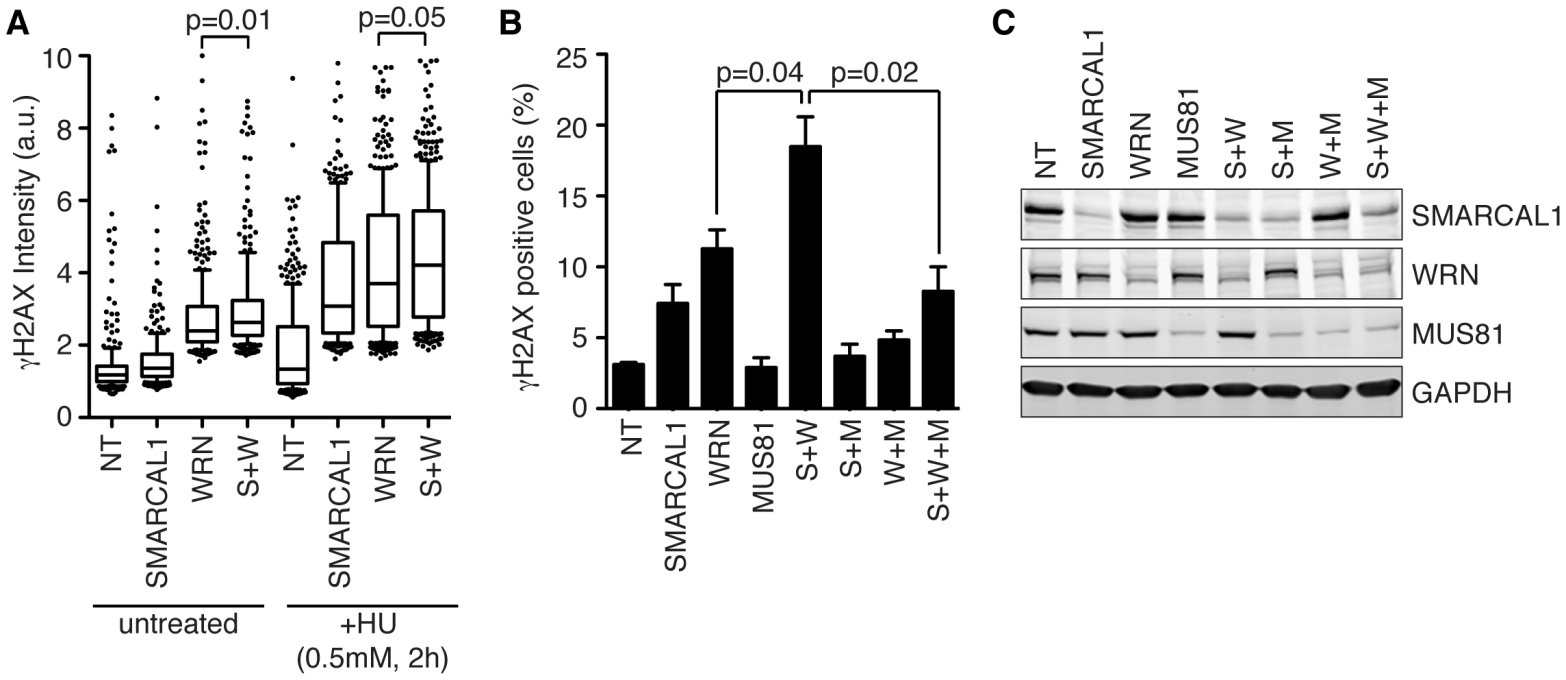
SMARCAL1 and WRN act independently to prevent MUS81-dependent double-strand breaks during S-phase. (A and B) U2OS cells were transfected with non-targeting (NT), SMARCAL1 (S), WRN (W), or MUS81 (M) siRNAs as indicated. (A) Cells were treated as indicated prior to staining with antibodies to γH2AX. γH2AX intensity per nucleus was determined by quantitative immunofluorescence using Cell Profiler software (Broad Institute). A box and whiskers plot with 10%–90% percentiles and outliers is shown. (B) Transfected cells were stained with antibodies to γH2AX and positive-staining cells were counted. Mean and SD are presented. (C) Lysates from transfected cells were separated by SDS-PAGE and immunoblotted with the indicated antibodies.

SMARCAL1 and WRN both act during DNA replication to promote the repair and restart of stalled replication forks. Consequently, SMARCAL1 and WRN deficient cells accumulate DNA damage during S-phase. Specifically, silencing either SMARCAL1 or WRN with RNA interference leads to the accumulation of γH2AX foci during S-phase even in unperturbed cells [2], [4], [9]. The γH2AX foci are likely sites of double-strand breaks since they are dependent on the action of the MUS81 structure specific endonuclease [9], [19]. As a genetic test of whether SMARCAL1 and WRN act in a common pathway to prevent MUS81-dependent DNA damage, we examined γH2AX phosphorylation in cells depleted of one or both of these proteins. As expected, silencing either SMARCAL1 or WRN leads to an increase in γH2AX phosphorylation, which is even further increased with addition of replication stress (Fig. 4A). The depletion of both SMARCAL1 and WRN at the same time leads to an even greater increase in γH2AX phosphorylation with and without HU treatment (Fig. 4A). The γH2AX phosphorylation in all circumstances is largely suppressed by MUS81 silencing (Fig. 4B). These results suggest that SMARCAL1 and WRN largely act in different pathways to suppress MUS81-induced DNA damage during S-phase.

**Figure 5:**
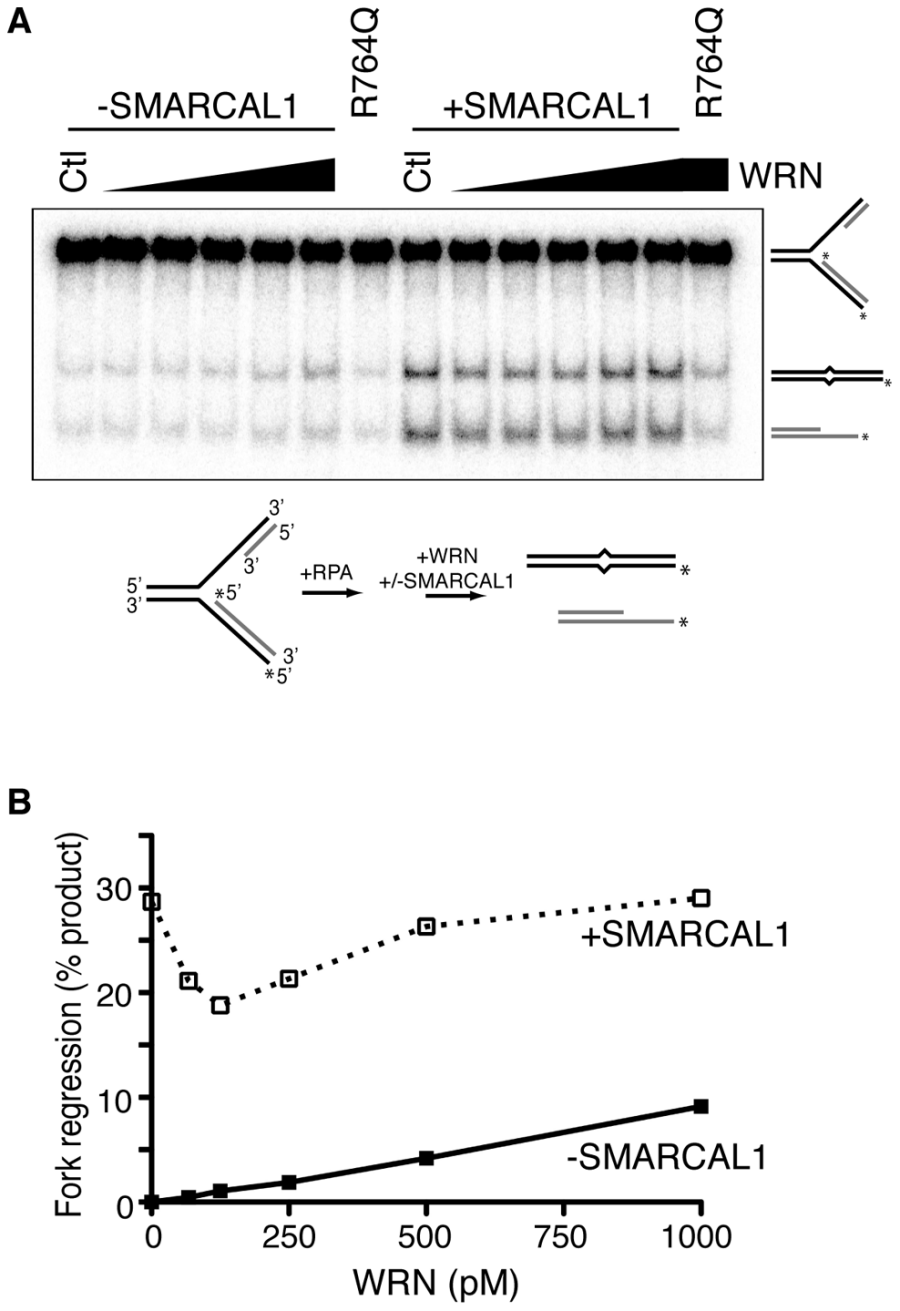
SMARCAL1 and WRN work independently to catalyze fork regression. Purified SMARCAL1 and WRN proteins were incubated with a ^32^P-labeled model replication fork substrate capable of undergoing fork regression (see supplemental table S1). The substrate contains a 30-nucleotide ssDNA gap on the leading strand. RPA was pre-bound to the substrate prior to the addition of enzymes. (A) 500pM SMARCAL1 was used where indicated. The Ctl reactions lacked WRN protein to show the amount of spontaneous fork regression without SMARCAL1 or the amount of fork regression in the presence of only SMARCAL1. R764Q indicates reactions with the ATPase deficient SMARCAL1 R764Q mutant as a control. (B) Quantitation of the percent of product formed in each reaction. The amount of spontaneous branch migration in the absence of any added protein was subtracted from each sample.

We next asked whether SMARCAL1 and WRN also act independently in a biochemical assay. Both proteins catalyze fork regression of model replication forks. However, their respective mechanism of action should rely on distinct intrinsic activities since WRN exhibits helicase activity while SMARCAL1 has been described as an annealing enzyme [18]. The cooperation of these activities to yield efficient fork regression is an attractive idea. Thus, we tested whether they could cooperate in a fork regression assay. A model replication fork was created by annealing oligonucleotides (Table S1). This substrate contains mismatches on the “template” strands to prevent spontaneous branch migration and a ssDNA gap on the leading strand template sufficient to bind one molecule of RPA in its high affinity binding mode. RPA was pre-bound to the DNA substrate. WRN has both helicase and exonuclease activities. To prevent degradation of the substrate, an exonuclease-deficient WRN protein was added to the reaction. As expected, increasing concentrations of WRN between 0–1000 pM led to modest but significant increases in fork regression (Fig. 5). At 500 pM WRN approximately 5% fork regression was observed. Addition of 500 pM SMARCAL1 in the absence of WRN yielded approximately 28% fork regression in identical conditions. When tested together with a constant 500 pM SMARCAL1 and increasing WRN concentrations from 0–1000 pM we initially observed a reduction in fork regression activity at low WRN concentrations followed by a steady increase in activity as the WRN concentration was increased. However, the combination of SMARCAL1 and WRN never yielded more activity than either protein alone. We interpret these results to indicate that at low WRN concentrations, WRN outcompetes SMARCAL1 for substrate binding, but since it has very low enzymatic activity compared to SMARCAL1, there is a substantial reduction in overall catalysis. At higher WRN concentrations, the amount of catalysis is increased in proportion to the activity of WRN concentration consistent with an independent contribution of SMARCAL1. We cannot rule out the possibility that SMARCAL1 and WRN work cooperatively on other substrates, but these data are consistent with an independent function of these two enzymes at stalled replication forks.

## Discussion

To understand how SMARCAL1 functions at stalled replication forks we purified SMARCAL1 protein complexes and identified interacting proteins by mass spectrometry. We identified several proteins complexed with SMARCAL1 including the WRN helicase/exonuclease. This is in agreement with previous mass spectrometry data [3]. We validated that WRN and SMARCAL1 are in a protein complex by reciprocal co-immunoprecipitation of endogenous proteins and co-localization at stalled replication forks. Further analysis indicated that the WRN and SMARCAL1 interaction is dependent on the single-stranded DNA binding protein RPA. WRN and SMARCAL1 bind to different surfaces on the RPA protein, and RPA acts as a scaffold allowing WRN and SMARCAL1 to associate.

While WRN and SMARCAL1 are found in the same complex, they appear to act largely independently. Their localization to stalled replication forks is independent of each other. In replicating cells, they both independently prevent MUS81-dependent γH2AX phosphorylation. Although γH2AX can mark many types of DNA lesions, the requirement of the structure-specific endonuclease MUS81 in its formation strongly suggests that in these cases it marks double-strand break formation. Finally, *in vitro*, they compete to bind model replication forks and work independently to remodel the fork.

Replication fork stalling is common during DNA replication [31], [32]. Since completing DNA replication is essential for cell viability, there are many mechanisms that have evolved to stabilize and repair stalled forks. Defects in any one of these mechanisms will not necessarily be catastrophic but will increase the chance of improper repair and generation of chromosomal abnormalities. Thus, inherited loss of function mutations in fork repair enzymes like WRN and SMARCAL1 cause diseases that include an increased predisposition to cancer [15], [16], [33]. While our data are consistent with a model in which these proteins function largely independently to catalyze fork repair and restart, we do not exclude the possibility that SMARCAL1 and WRN could work cooperatively in specific circumstances. For example, WRN functions at telomeres and at common fragile sites [34], [35], so it would be useful to examine whether SMARCAL1 also functions at these genomic locations. An alternative hypothesis is that SMARCAL1 might function with the WRN exonuclease activity to repair stalled DNA replication forks in specific circumstances.

## Supporting Information

Table S1 **Oligonucleotide sequences used to make fork reversal substrates.**
(TIF)
