# Supplementary figures and images for: Identification and Characterization of SMARCAL1 Protein Complexes
Source: PLoS One. 2013 May 9;8(5):e63149. doi: 10.1371/journal.pone.0063149 (PMC3650004; doi:10.1371/journal.pone.0063149)

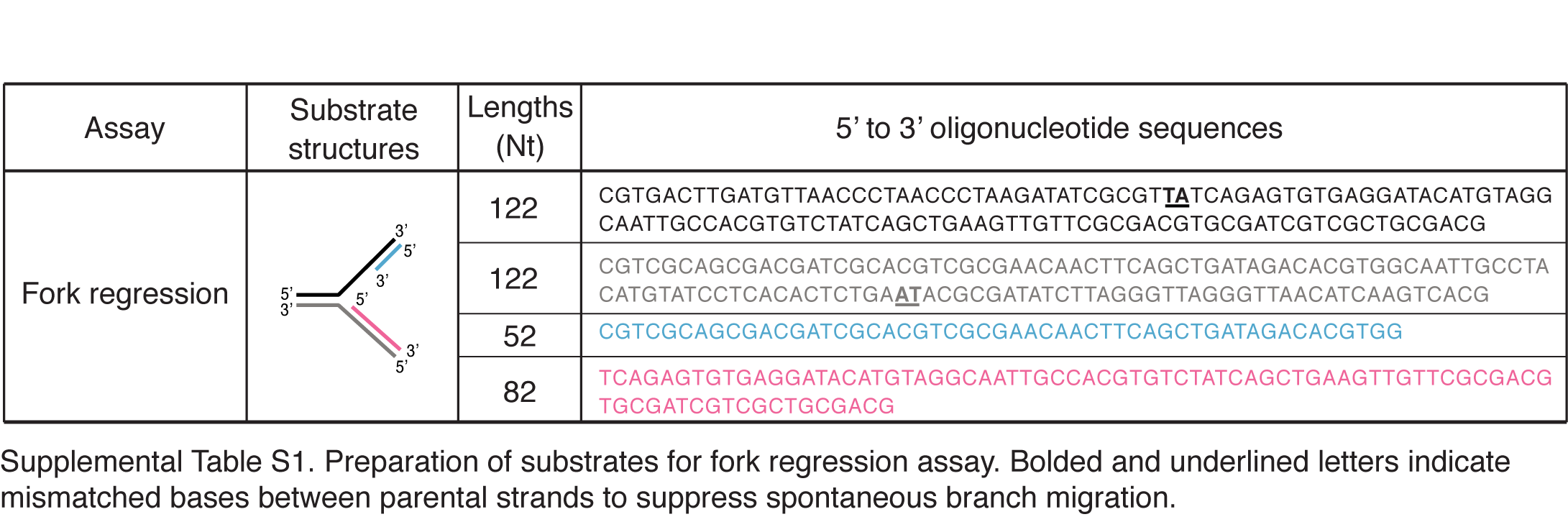

Supplement: Table S1 — Oligonucleotide sequences used to make fork reversal substrates. (TIF) [file pone.0063149.s001.tif]
